# Evaluate the effects of platelet rich plasma (PRP) and zinc oxide ointment on skin wound healing

**DOI:** 10.1016/j.amsu.2018.11.009

**Published:** 2018-12-04

**Authors:** Barham Jalal Abdullah, Nazmi Atasoy, Abdullah Khalid Omer

**Affiliations:** a Sulaimani Veterinary Directorate, Veterinary Quarantine, Sulaimani International Airport, Iraq; b University of Van Yȕzȕncȕ Yıl, Veterinary College, Department of Surgery, Van, Turkey; c Sulaimani Veterinary Directorate, Veterinary Quarantine, Bashmakh International Border, Iraq

**Keywords:** Platelet rich plasma, Inflammation, Zinc oxide, Skin wound healing, Rabbits

## Abstract

In this study efficiency of platelet rich plasma (PRP) and zinc oxide on full thickness wounds created on rabbits was researched. This study conducted on 24 New Zealand rabbits divided 2 groups. A circular of 1.5 × 1.5 cm (2.5 cm^2^) full thickness skin wound was created under the general anesthesia. 1 ml PRP (5.503106/mm^3^) was applied to the one of the wounds subcutaneously. To the other wound 1 mm^3^ zinc oxide ointment was applied once a day during the study. Wound contraction rates were measured, biopsy materials were collected and evaluated macroscopically and histopathologically postoperatively 3rd, 7th and 15th days. As a result it is determined that PRP and zinc oxide accelerated wound contraction rates between the groups were determined as 3rd day p = 0.007, 7th day p = 0.0002 and 15th day p = 0.002.

## Highlights

• Wound healing is inborn to all species and is the biologic procedure by which the body repairs itself after injury, whichever it be traumatic, complicated, infected and/or surgical.
• Platelet rich plasma (PRP) gel is measured to be progressive wound treatment for acute and chronic wounds. PRP gel has mainly been applied to improve or accelerate healing of wound.
• Zinc is a vital trace element in the human body and its significance in wellbeing and infection is valued. Zinc deficiency of genetic or dietary cause can prompt neurotic, pathologic and physiological changes and deferred wound healing.
• Topical treatment with autologous PRP can be used as clinical therapy and can enhance tissue healing and enhanced angiogenesis compared to zinc oxide treatments.

## 1 Introduction

Wounds are characterized as skin deformities created by electrical, thermal, chemical and mechanical damages that outcome in an opening or damaging the integrity of the skin, or by the occurrence of a fundamental therapeutic or physical issue, or may likewise be characterized as the disturbance of anatomical and physiological integrity of living tissue [[1], [2], [3]]. Wound healing is inborn to all species and is the biologic procedure by which the body repairs itself after injury, whether it be traumatic, complicated, infected and/or surgical [4,5]. Wounds are corporeal or bodily damage that outcome in a disruption or rupture in the skin and leads to an interruption in the natural skin anatomical structures and physiological functions as described by wound healing society (WHS) [6,7].

Wound healing procedure is managed by a grouping of occasions, including coagulation, inflammation, production of granulation tissue, epithelialization, and tissue renovation [8]. Wound curative biotic procedure is intervened by connecting sub-atomic signs, principally on the part of growth factors (GFs) and cytokines which fortify and moderate the chief cellular actions underlying healing [9,10], and also mediated by several proteins, including many growth factors such as, epithelial growth factor (EGF), fibroblast growth factor (FGF), and platelet-derived growth factors (PDGF) [10,11].

In both human and animals, wound healing depend on some factors including blood supply, size of wound, pressure and movement of wound edges, individual's susceptibility to infection, kind and state of basic tissue, additionally age, malnourishment, illness (for example, diabetes mellitus and cancer), kind of injury, and tissue perfusion can adversely impact the wound healing process [10,12].

Wound healing is an active procedure consists of four phases that are connecting with each other's, including hemostasis, inflammatory, proliferative and remodeling phases [4,12,13]. The early stage in the wound curing procedure is the inflammatory phase, which is viewed as a basic step of the wound curing procedure, crucial for avoiding disease, and included in tissue regeneration [14]. On one occasion a wound starts curing, usually the procedure determines with complete injury termination. Nevertheless, healing of chronic and acute wounds can become diminished by patient reasons or causes (for example, diabetes) and/or factors affecting wounds (for example, contamination or infection) and also obesity, sex hormones, stress, smoking, etc. [12,15]. Resuming a wound with diminished curing is problematic since good standard wound care does not continuously provide a developed healing outcome and frequently further advanced managements are employed [[16], [17], [18]].

Platelet rich plasma (PRP) gel is measured to be progressive wound treatment for acute and chronic wounds. The clinical and experimental use of platelet rich plasma (PRP) has been verified, to become a satisfactory clinical treatment for soft tissue healing and for numerous or various orthopedic trauma, dental and plastic surgical applications, and PRP gel has mainly been applied to improve or accelerate healing of wound [18,19].

“PRP contains various growth factors, proteins and peptides, chemokines, cytokines and a fibrin scaffolding obtained from a blood of patient [20,21], epidermal growth factor (EGF), insulin-like growth factor (IGF), interleukin-1 (IL-1), platelet factor 4, platelet derived growth factor (PDGF), vascular endothelial growth factor (VEGF), transforming growth factor-β (TGF-β), platelet-derived angiogenesis factor, platelet derived endothelial growth factor, epithelial cell growth factor, fibrinogen, fibronectin, osteonectin, osteocalcin, vitronectin, and thrombospondin that are discharged from the granules of platelets upon their actuation by means of the expansion of thrombin” [13,22,23].

“Platelet determined arrangements contain both anti and pro-inflammatory cytokines that can produce a tough pro-inflammatory response somewhat in charge of activating wound repair” [22,24,25]. It was recently showed by El Backly et al. [26], that a platelet rich plasma-based layer intended to substitute the periosteum could make an angiogenic and osteogenic microenvironment qualified for activating endogenous regenerative components and prompting general improvement of bone recovery.

The special effects of PRP have mainly been established in different works, in both healing and nonhealing wounds [[27], [28], [29], [30], [31]] in chronic and acute wounds [28] in animals [22,32,33] and humans [34,35].

Application of Platelet-rich plasma (PRP) has been used to quicken wound healing [15,36]. PRP is the plasma portion, which is typically arranged from autologous blood and has been utilized to manage both chronic and acute wounds [15,37]. Additionally, a few researches have established that PRP has antimicrobial action against *Candida albicans*, *Cryptococcus neoformans*, *Escherichia coli* and *Staphylococcus aureus*, but there were no effects on some bacteria for example, *Klebsiella pneumoniae, Enterococcus faecalis,* and *Pseudomonas aeruginosa* [38,39].

Zinc is a vital trace element in the human body and its significance in wellbeing and infection is valued [40]. Zinc deficiency of genetic or dietary cause can prompt neurotic, pathologic and physiological changes and deferred wound healing [40]. Recently zinc (Zn) due to various biophysiological capacities, increase significant enthusiasm or attention for wound consideration [41].

Topical zinc treatment decreases wound remains and progresses epithelialization in surgical lesions in the rat [42,43]. Comments that topical zinc prompted decrease of wound remains and necrotic material injuries of various etiologies [[43], [44], [45]]. The purpose of this study was to investigate and evaluate with comparison the enhancing effect of PRP and zinc oxide as clinical use for the topical treatment of skin wound healing in rabbits.

## 2 Materials and methods

### 2.1 Materials

#### 2.1.1 Animals

All experimental protocols were approved by the local animal care committee in agreement with Faculty of Sulaimani Veterinary Medicine office regulations (2016/03). In the present study, twenty-four New Zealand-white rabbits, weighting about 1.00–1.5 kg with averagely 8 weeks’ old were randomly divided into two groups based on treatment each group containing 12 animals.

#### 2.1.2 Equipments and chemicals

All measurements were performed by using Routine surgical kit, xylazine hydrochloride (Rompun, Bayer), ketamine hydrochloride (Ketasol, Interhas), platelet-rich plasma (PRP), zinc oxide ointment B-Laboratory Testing.

### 2.2 Methods

#### 2.2.1 Surgical procedure

All rabbits were anesthetized via intramuscular injection xylazine (3 mg/kg body weight) and ketamine (30 mg/kg body weight) combination. Prior to the experiment, the animals were accustomed to environment about one week and all animals were fasted for 12 h before the operation. A circular of (1.5 × 1.5 cm) full-thickness skin wound was created under aseptic conditions on waist hip of rabbits. Wound areas were calculated as 2.5 cm^2^ by measuring the edges of the wound at the first day by digital vernier caliper.

#### 2.2.2 Treatment of wounds

Treatment of wounds by PRP and zinc oxide were performed, immediately following the incision wound creation, 1 ml PRP (5.503106/mm^3^) was used on one wound just once along the wounds time, and 1 mm^3^ zinc oxide was applied once a day to another wounds till the end of selected days in this study. On the third, seventh and fifteenth days, the wound area was measured by stainless steel digital vernier caliper 150 mm (6") from (Ningbo Faith Safe Protection CO., LTD) and comparison between zinc oxide and PRP treatment was done. After finishing the treatment time in 3rd, 7th and 15th days, tissue samples were taken from each animal wounds for microscopical findings took biopsy by punch techniques, full-thickness lesion specimen (far from 5 mm wound edge) were cut precisely then kept on 10% buffered formalin when remains for two days in each steps until fixed and removed of debridement, followed putted on tissue block and sent to a pathology laboratory for histopathological examination directly after third, seventh and fifteenth days. The samples were processed with routine histologic procedures are then detected in 10% buffered formalin, embedded in paraffin, sectioned transversely (4 μm thick). Then, transverse sections were stained with hematoxylin and eosin (H and E) stain for histopathological examinations.

#### 2.2.3 Preparation of PRP

Three milliliters of homologous fresh blood were obtained in a sterile tube from the heart of each rabbit, by cardiac puncture technique, then treated with 1 mL of heparin, anticoagulant sodium. Heparin tube is used for separating plasma after blood collection, turn the heparin tube upside down with 180° and shake 8–10 times at room temperature 25 °C. After that blood was centrifuged immediately at 3200 rpm for 15 min (1600 g). Blood was separated into three layers according to the whole blood by gravity after centrifugation, red blood cells at the bottom, acellular plasma in the supernatant, which is the upper layer, and platelets and leukocytes which is the “buffy coat” layer in between. The upper layer was transferred with a sterile pipette to another 10 mL centrifuge tube without buffy coat and re-centrifuged at 3200 rpm for 15 min (1600 g) (Httich Zentrifugen, Werk Nr, Germany). About 1.0 mL of PRP (platelet count about 5.503106/mm3) was pipetted from the bottom of the tube and about 1.5 mL of platelet-poor plasma (PPP) was harvested as a supernatant. The prepared PRP, PPP, and whole blood of the rabbits were subjected to platelet and PPP was added a little at a time to adjust PRP to different platelet concentrations: low-concentrated PRP (platelet count 23106/mm3) and high concentrated PRP (platelet count 53106/mm3). Finally, each of activated PRP was prepared as a total amount of 0.5 mL, which contains a high concentration of platelets [46,47].

### 2.3 Statistical analysis

Statistical analyses were performed on graphed prism 6.01 software. ANOVA test was applied to analysis the significant differences in both PRP and zinc oxide groups. Duncan (Kruskal-Wallis) test was done to compare between groups. Differences were considered significant when the P values was = 0.0001.

## 3 Results

### 3.1 Macroscopic findings

**Fig. 1:**
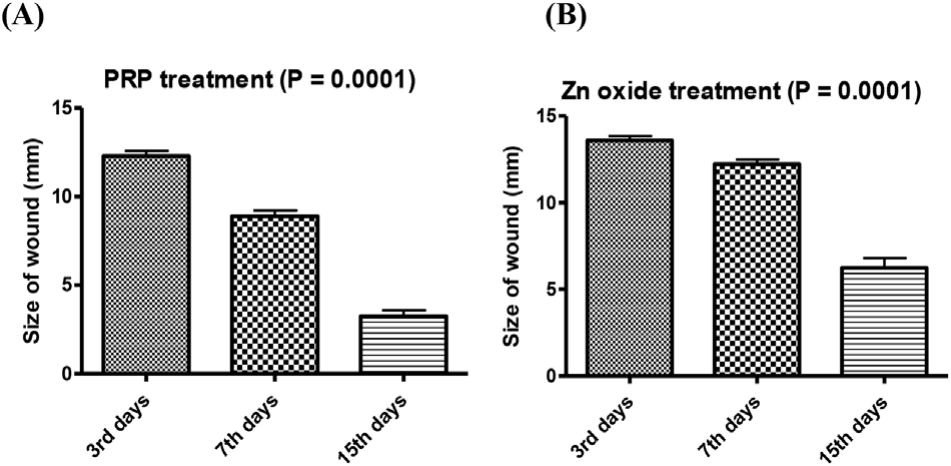
Size of wound healing by PRP (A) and zinc oxide (B) treatment (P = 0.0001).

No infection was observed in all animals during the experimental period of this study. A circular full-thickness wound of 2.5 cm^2^ (15 mm) diameter was created on the median line in the waist-hip ratio of the experimental rabbits. Our results indicated a high significant differences (P = 0.0001) between PRP and zinc oxide treatments with high significant decreases in size of wound healing (Fig. 1).

**Fig. 2:**
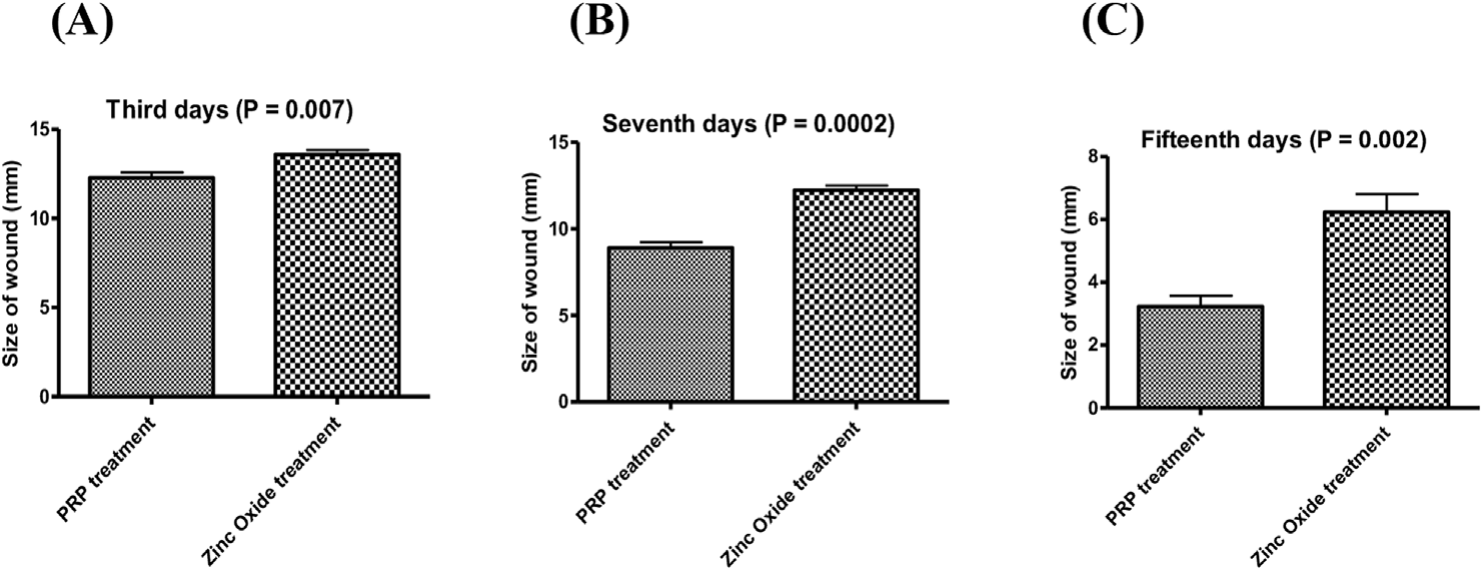
Size of wound after healing by both PRP and zinc oxide treatment, during 3rd days (P = 0.007), 7th days (P = 0.0002) and 15th days (P = 0.002).

There is a significant difference between PRP treatment and zinc oxide treatment p value = 0.007 in third day, also there is significant difference between PRP treatment and zinc oxide treatment p value = 0.0002 in seventh days and there is significant difference between PRP treatment and zinc oxide treatment p value = 0.002 in fifteenth days as shown in (Fig. 2A and B and C).

**Fig. 3:**
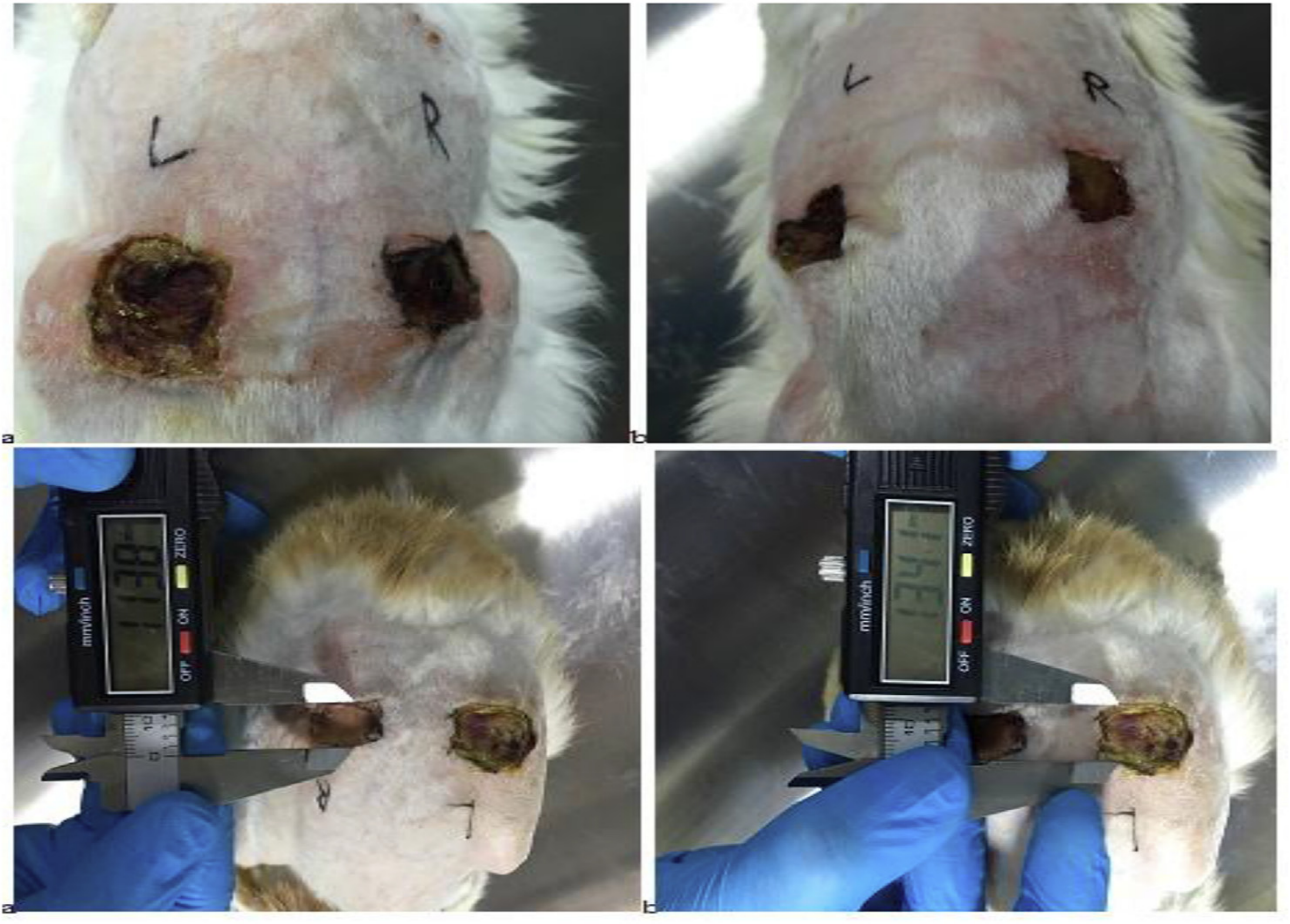
Wound size 3 days after platelet-rich plasma (PRP) (a) and zinc oxide (b) treatment.

Even though all group of rabbits were in great and healthy situation and showed no sign and symptoms of infection on day 3 in the presence of PRP and zinc oxide management, the wound showed slow propensity to heal. Scratching in all groups had just begun, crusting rate was more advanced and improved in the PRP group and wound size decreased about 3.62 mm/inch (0.362 cm) compared to zinc oxide that healed only about 1.59 mm/inch (0.159 cm) (Fig. 3).

**Fig. 4:**
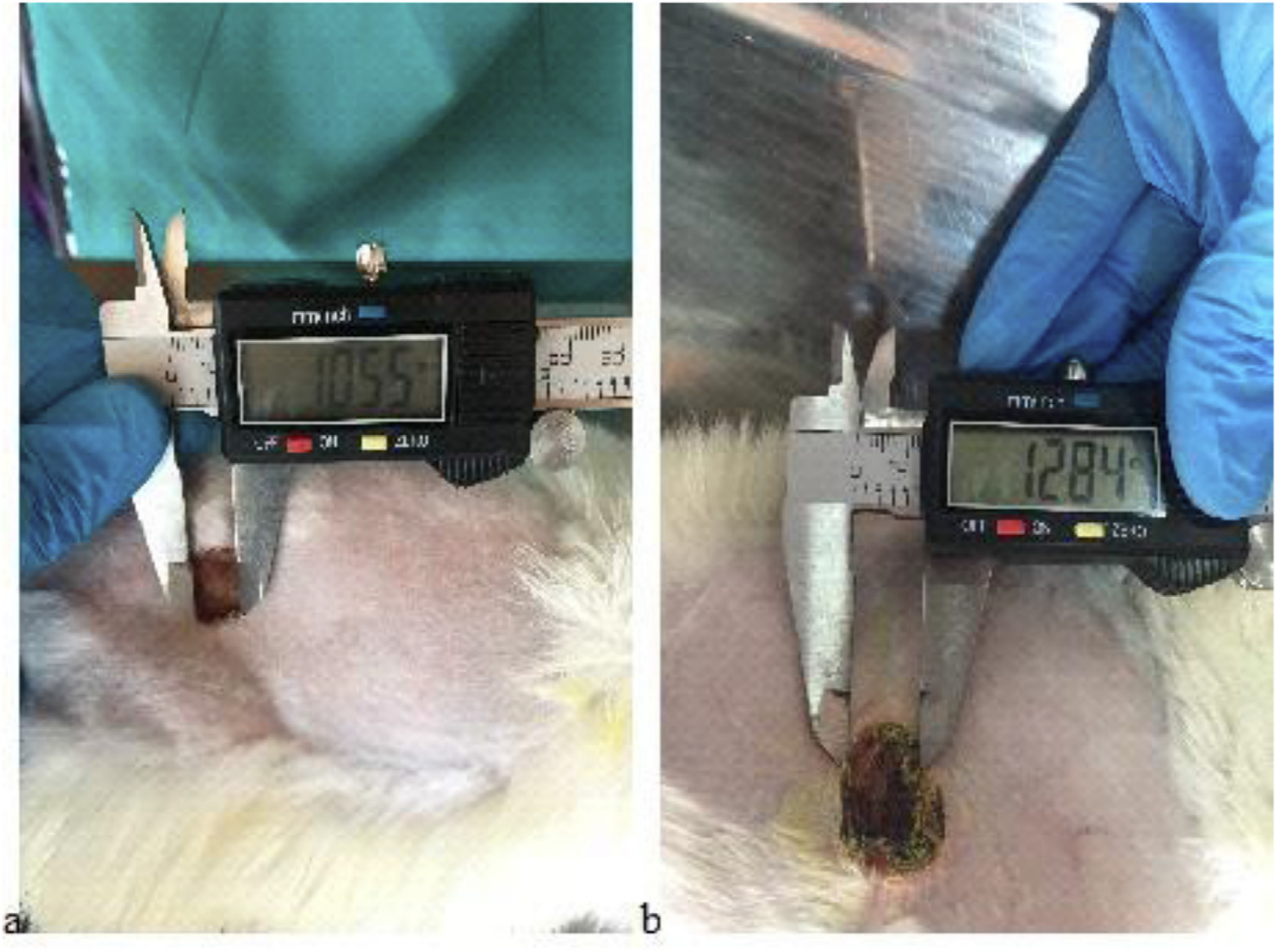
Wound size 7 days after platelet-rich plasma (PRP) (a) and zinc oxide (b) treatment.

On the day 3, formation of newly formed vascularized tissue was seen in two groups. Although partial complete crusting in the day 7 by PRP group showed an increase 4.45 mm/inch (0.445 cm) in the crusting rate than in zinc oxide 2.16 mm/inch (0.216 cm) crusting group. Both PRP and zinc oxide groups contain no infection and congestion, and then shown no exudate, but small irregularities in the wound edges were seen in zinc oxide group managements (Fig. 4).

**Fig. 5:**
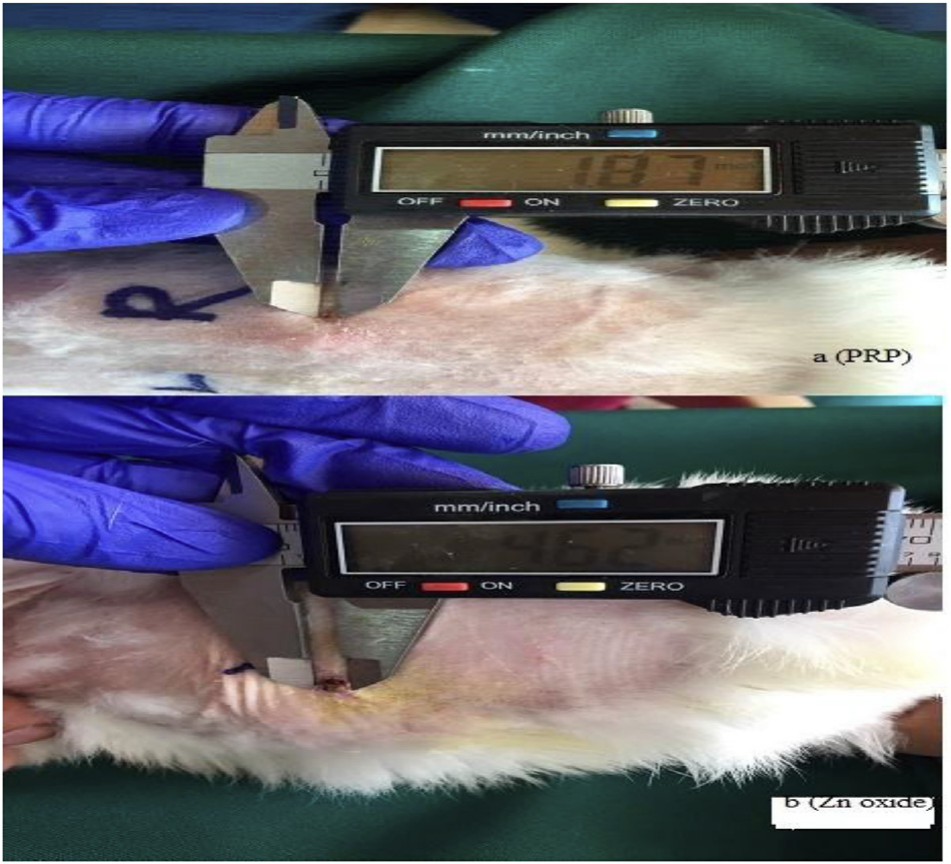
Wound size 15 days after platelet-rich plasma (PRP) (a) and zinc oxide (b) treatment.

On the day 15, the wound appeared complete healing, the newly formed tissue adhered well to the subcutaneous tissue, even at the edges of the wound it was observed full improvement in the group PRP starts to slow down compared to the group of zinc oxide, scabbing as faster in both PRP and zinc oxide groups and steadily progressing healed were observed. Both PRP and zinc oxide groups contain no infection or contaminations. Complete healing 13.13 mm/inch (1.3 cm) was seen under PRP management compared to zinc oxide 10.38 mm/inch (1.03 cm) group treatment. In zinc oxide group the wound appeared incomplete clinically and still remained for healing completely (Fig. 5).

### 3.2 Microscopic findings (histopathologic findings)

**Fig. 6:**
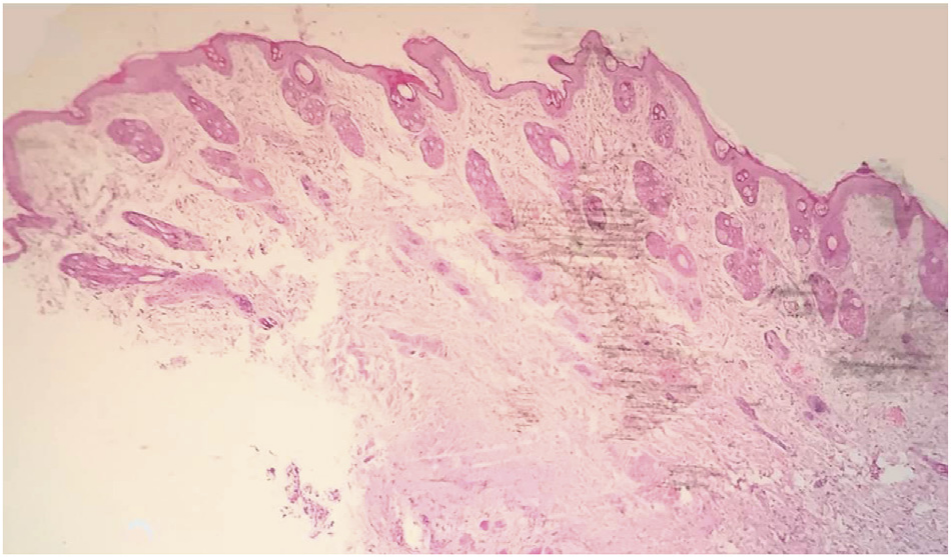
Light micrograph shows normal histological skin structures in rabbit (H and E stain, 40X).

The microscopic observation affirmed the aseptic conditions throughout the study in the incised wound in both groups. The histological structure of skin was showed normally that has composed of the epidermis, dermis and hypodermis as in (Fig. 6).

**Fig. 7:**
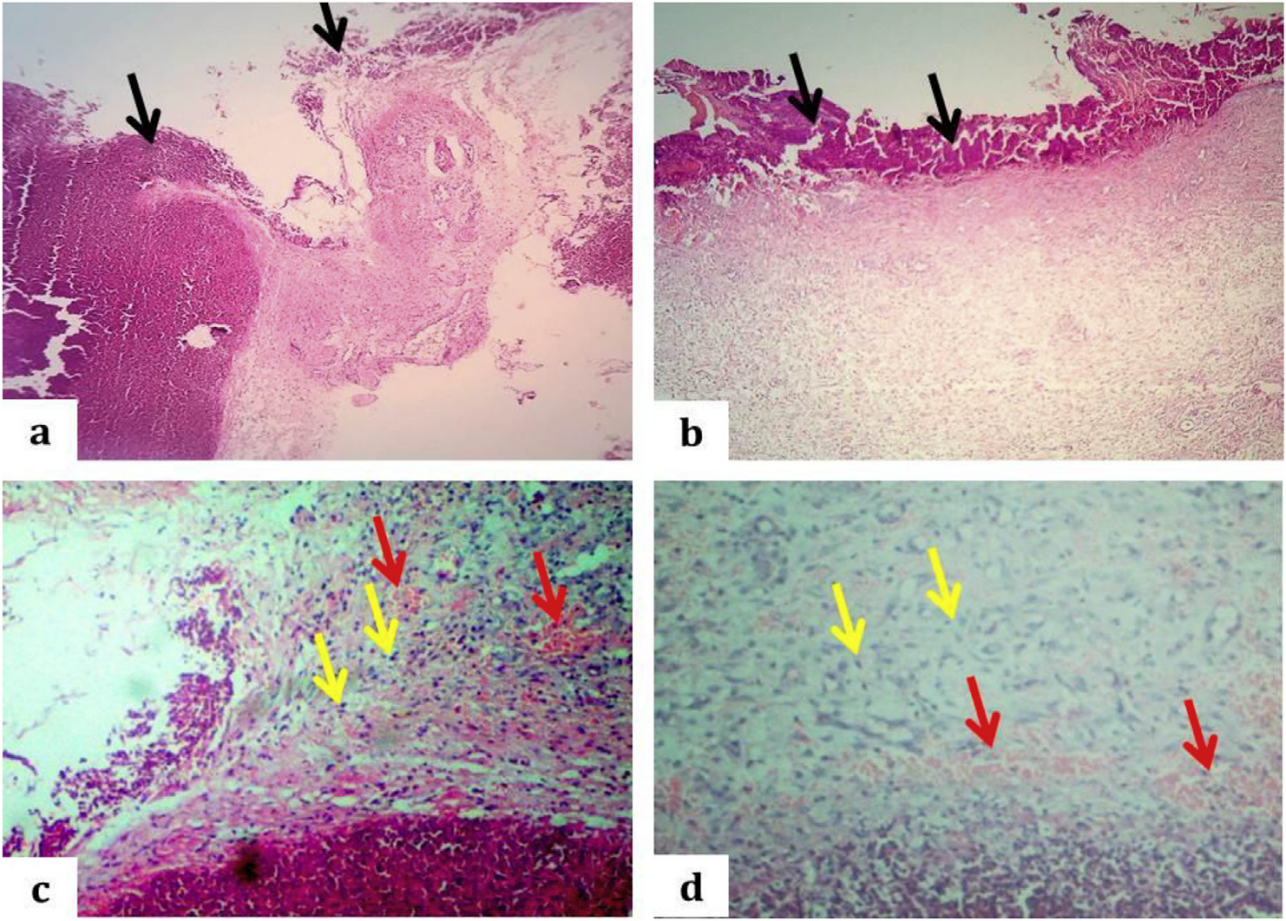
Light micrograph of incised wound of the rabbit's skin after 3 days. a: The open edges wound with large amount of necrotic debris and fibrin within the incised gap and cover the wound surface as showed by black arrows in zinc oxide group, (H and E stain, 40X). B: The partially closed wound with moderate amount of necrotic debris with fibrin that cover the wound surface as showed by black arrows in PRP group, (H and E stain, 40X). c: Granulation tissue: Slight infiltration of neutrophil (yellow arrows) with underlining neovascularization as indicated by red arrows in zinc oxide group (H and E stain, 100X). d: Granulation tissue: Moderate infiltration of neutrophil (yellow arrows) with underlining neovascularization as indicated by red arrows in the PRP group (H and E stain, 100X).

Through the inflammatory and proliferative stage, after 3 days, in the zinc oxide group, wound showed incision gap (not closed well) with large amount of necrotic debris and fibrin cover the surface of wound also within the wound gap, with few underlying neovascularization and infiltration of few numbers of inflammatory cells mainly neutrophil (granulation tissue), and the epithelial surface still absent in this period (Fig. 7 a, c), while, in the PRP group the covering epithelial cells proliferate and 'crawl' atop the wound bed, given that cover for the new tissue with underlying markedly neovascularization and considerable number of inflammatory cells mainly neutrophil (granulation tissue) as in (Fig. 7 b, d).

**Fig. 8:**
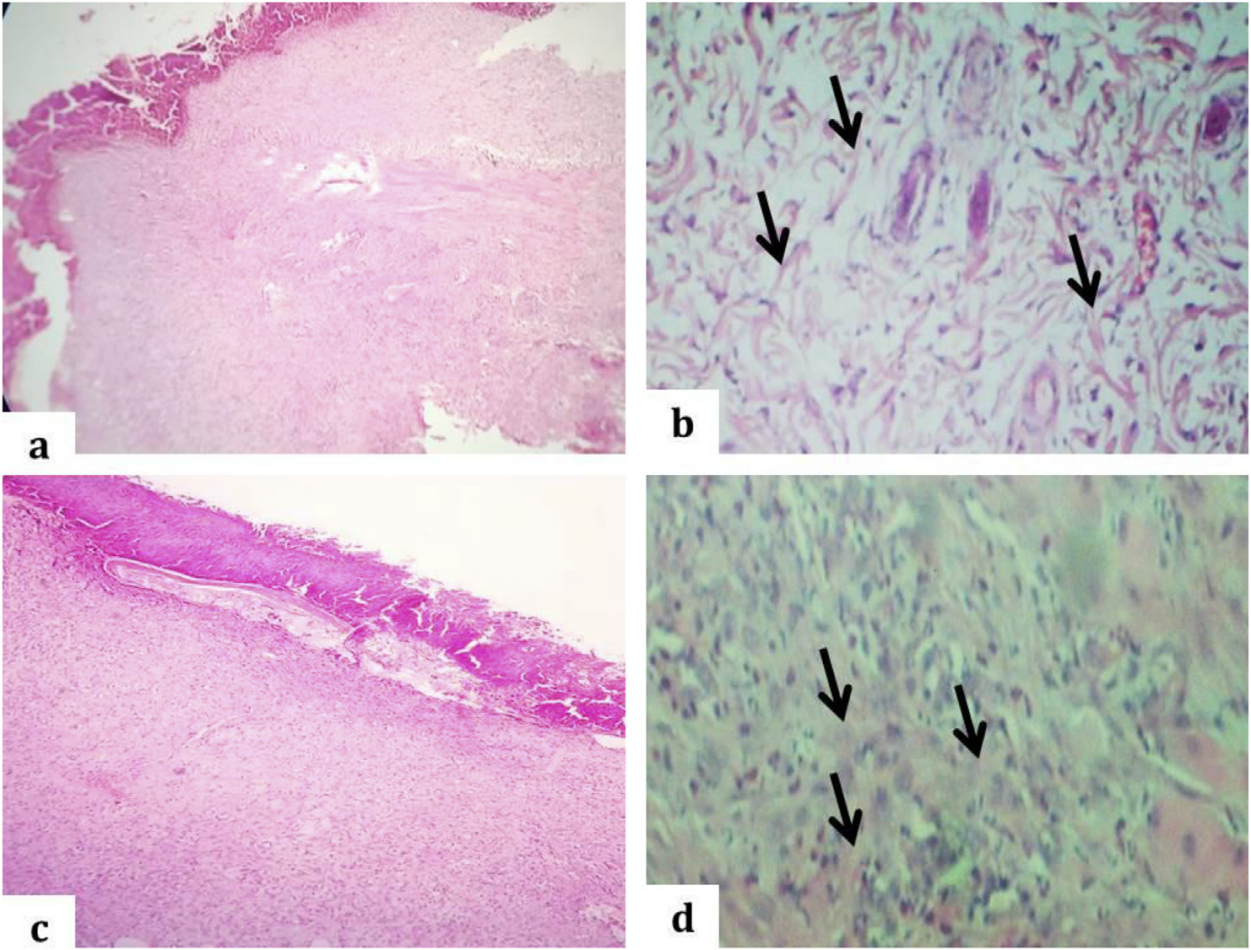
Light micrograph of incised wound of the rabbit's skin after 7 days. a: The incomplete re-epithelization with underlying granulation tissue formation in zinc oxide group, (H and E stain, 40X). b: Dermal matrix show few thin bundle of collagen fibers admixed with inflammatory cells as showed by black arrows in zinc oxide group, (H and E stain, 100X). c: The complete re-epithelization with intact stratum corneum in PRP group, (H and E stain, 40X). d: Thickening of dermal matrix due to presence of bundle of collagen fibers intermingled with fibroblast and few inflammatory cells as indicated by black arrows in the PRP group (H and E stain, 100X).

During the proliferative stage after 7 days, the zinc oxide group showed incomplete re-epithelization with underlying granulation tissue formation, the dermal cell proliferation with matrix accumulation and deep contraction of the wound that leads to wound rising. Presence of fibroplasia with thin bundle of collagen fiber (Fig. 8a and b), while the PRP treated group showed complete re-epithelialization with an underlying considerable number of inflammatory cells, mainly neutrophil and matrix accumulation like presence of thick bundle of collagen fiber when compared to zinc oxide group and deep contraction that is leads to wound rising and complete wound process (Fig. 8c and d).

**Fig. 9:**
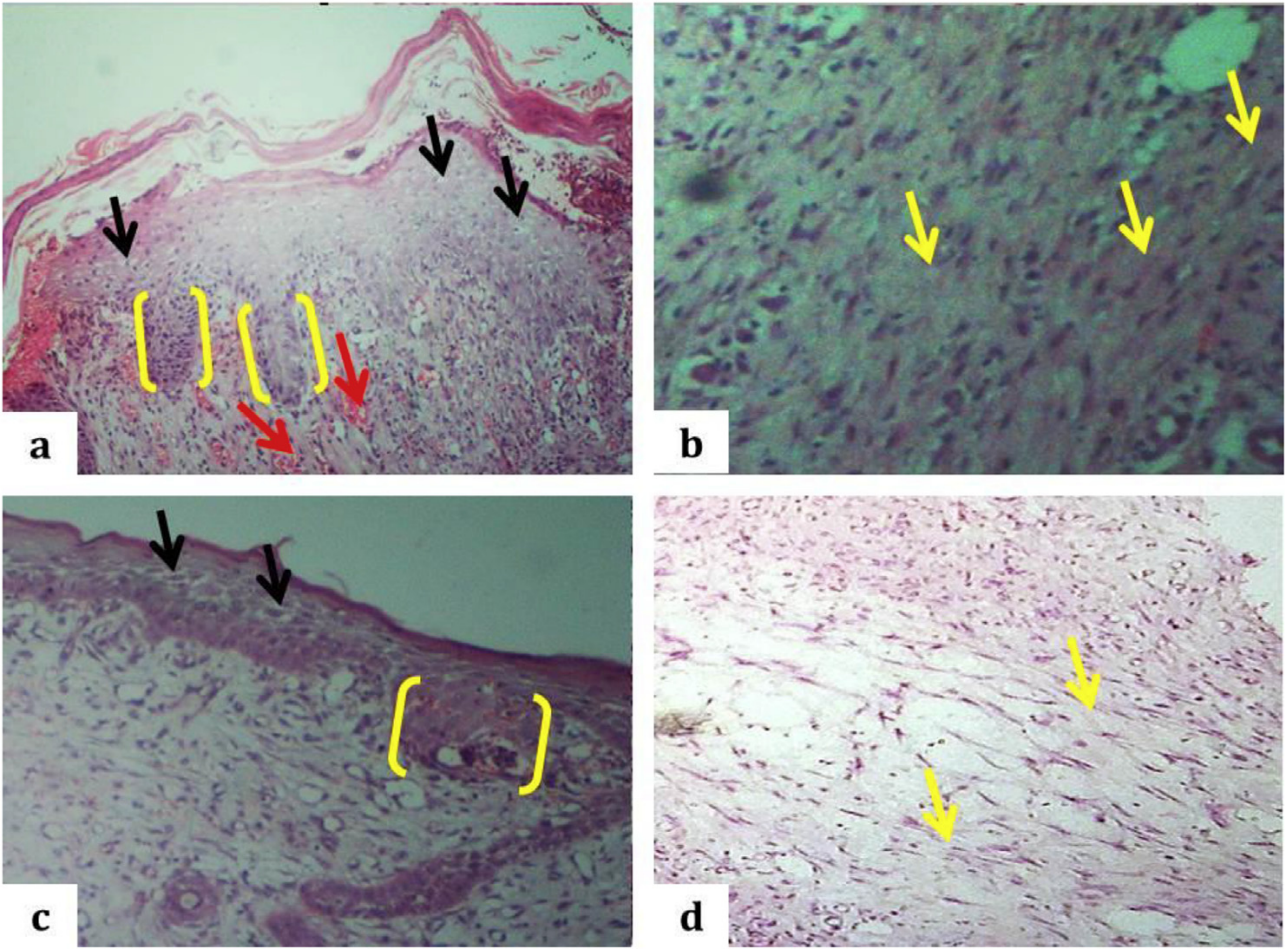
Light micrograph of incised wound of the rabbit's skin after 15 days. a: The complete re-epithelization without intact stratum corneum, presence of keratohyalin cells (black arrows), the rete ridges (yellow right brackets) and neovascularization as indicated by red arrows in zinc oxide group, (H and E stain, 40X). b: Underlying neovascularization with considerable fibroplasia admixed with collagen bundles as showed by yellow arrows in zinc oxide group, (H and E stain, 100X). c: The complete re-epithelization with intact stratum corneum, presence of clear keratohyalin (black arrows), the rete ridges (yellow right brackets) in PRP group, (H and E stain, 40X). d: Fibrous collagen + tissue with fibroblast as indicated by yellow arrows in the PRP group (H and E stain, 100X).

During the wound contraction stage about 15 days after, the zinc oxide treated group showed complete epithelization with the rete ridge formation but still showed a hyperplastic irregular epidermis without stratum corneum with keratohyaline granules (the stratum corneum not intact), with underlying granulation tissue formation characterized by fibroblast and collagen perpendicular to new blood vessel and parallel to wound surface (Fig. 9a and b). Furthermore, PRP treated groups showed completing the process of re-epithelization with rete ridge formation and intact stratum corneum, with clear keratohyaline granules, decreasing cellularity and absence neovascularization underlying fibrous tissue; fibroblast and collagen bundles parallel to the wound surface. The newly formed dermis becomes greatly thicker, with a base layer of organized collagens and the accumulation of immature matrix as wound filler. Matrix accumulation and remodeling lead to a strong reduction of the scar area, it has an appearance like normal skin structure (Fig. 9c and d).

## 4 Discussion and conclusion

Wang et al. [48] reported the anesthetic protocol in a study conducted on rabbits at 50 mg/kg ketamine hydrochloride and 5 mg/kg of xylazine hydrochloride. Also Paradhan et al. [49] showed by twenty-two New Zealand rabbits as anesthetics in their study of 25 mg/kg of ketamine hydrochloride and 3 mg/kg in the report that they have achieved successful anesthesia by xylazine hydrochloride. In this study all animals were anesthetized with intramuscular 3 mg/kg Xylazine HCI and 30 mg/kg Ketamine HCI combination and no complications were seen about anesthesia protocol.

Wounds do not follow the normal healing in the presence of inadequate amount or lack and deficiency of growth factors [50,51] angiogenic response [52], macrophage dysfunction, the amount of phenotype [53], collagen amount and the resulting granulation tissue character [54], fibroblast, keratinocytes migration and improvements [55]. The positive role of platelets in normal wound healing showed by different clinical researches, according to our results PRP and zinc oxide when locally applied can hasten and promote normal wound healing [10,[28], [29], [30], [31]]. PRP contains various growth factors that play an important role in the treatment of normal wound healing and their effects on tissue regeneration have mainly been established in various parts of the medicine, for instance, dentistry, oral implantology, orthopedics, sports medicine and the management of skin disorders [12,56].

In this study after 3 days of treatment by both PPR and zinc oxide, there were significant differences between PRP and zinc oxide treatments (Fig. 2-A) statistically (P = 0.007) on third day treatments. Comparison between these two treatments, PRP is more effective on healing. The significant impacts of PRP are obtains from PDGF, which has been distinguished as a critical protein for hard and soft tissue repairing. PDGF has been appeared to empower chemotaxis, mitogenesis and the replication of immature microorganisms at the site of an injury to the site of tissue damage [57]. PDGF additionally fortifies the creation of fibronectin, a cell adhesion molecule used in cellular proliferation and migration amid repairing, including osteoconduction and hyaluronic acid, and it helps in inducing wound contraction and remodeling [58].

During 7 days of treatments, our results indicated high effect of both PRP and zinc oxide on healing, but PRP is more effective if compared with zinc oxide. There was a high significant (P = 0.0002) differences (Fig. 2-B) between both PRP and zinc oxide statistically, and these results show a significant decrease in size of wounds. As of late, the utilization of PRP has been proposed as a method for acquiring high centralizations of growth factors required in tissue curing, healing and recovery [57]. It has been reported that PRP application is significantly increased the acceleration of the both hard and soft tissue healing in humans [59].

At the time of 15 days’ treatments by PRP and zinc oxide indicate high effects on wound healing, PRP was more effective while zinc oxide was less if compared with PRP treatments. Complete healing of wound was observed in PRP group at 15 days of treatment. There was a high significant decrease of the wound size (P = 0.002) (Fig. 2-C) between both PRP and zinc oxide treatments. Also within PRP treatments our results indicate a high significance decrease of the wound size (Fig. 2-A, B and C) on each third, seventh and fifteenth days and at last (15th) days of experimental and clinical analysis for both PRP and zinc oxide treatments we have a high significant decrease and complete healing was done for PRP treatment and partial complete healing for zinc oxide treatment (Fig. 2). Similar results were found by Ostvar et al. [13], who used PRP during 7, 14 and 21 days of treatment and show a significant decrease of wound size if compared with non-PRP group treatments in rabbits.

Complete healing of the wound was observed about 15 days after the PRP use for single dose and also partial complete healing was viewed by zinc oxide groups, this results confirm the effects of PRP was more than zinc oxide. The growth factors existing in PRP is fit for shaping fibrin coagulation, advancing fibroblast multiplication and up-directing collagen amalgamation in the ECM. In this manner, the utilization of PRP at damage locales may have the capacity to advance wound repairing [57]. The results of this researches show a positive role of PRP and zinc oxide to accelerate wound healing, similar positive roles of PRP are found by Iacopetti et al. [10], Ostvar et al. [13] on cutaneous or skin wound healing in horse and rabbit respectively.

Strong effect of PRP was reported by Alissa et al. [59] and shows statistically significant improvement in soft and bone tissue healing. Celio-Mariano et al. [60] find strong effect of PRP and publicized significant development in bone healing in PRP sited therapy compared to non-PRP treated sites. Recently the positive effect of PRP on bone regeneration is study and acceleration with enhance bone regeneration is found at the time of direct application of PRP sideways the mandibular fracture lines [61].

Crovetti et al. [28] report good results of PRP gel in healing cutaneous chronic wounds with different origin and etiologies including diabetic, vascular insufficiency, vasculitis, neuropathic, post-traumatic and infectious disease. Improve healing of tissue at the cell level through the enlistment proliferation is recommend by Ahmad et al. [62]. Normal tissue regeneration by PRP is shows by Shin et al. [63].

Keefer et al. [43] describe that topical zinc treatment diminishes wound remains and enhances epithelialization in surgical wounds in the rat. Our zinc oxide results resemble these similarities. It was reviewed by Lansdown [42] and reported by Keefer et al. [43] that topical zinc oxide therapy decreases wound debris and improve epithelialization in surgical wounds in the rat. Agren et al. [64] demonstrate that topical zinc oxide therapy improves re-epithelialization of incomplete thickness wounds in nutritionally stable pigs and that the method of conveyance of zinc is presumably basic for accomplishing the gainful healing impact of zinc.

This study suggests that topical treatment with autologous PRP can be used as clinical therapy and can enhance tissue healing and enhanced angiogenesis compared to zinc oxide treatments. It can be concluded that PRP results with both macroscopical and microscopical data analysis discovered that wound healing time of PRP therapy group was shorter than that of zinc oxide therapy, and complete re-epithelization was done in PRP groups compared to zinc oxide groups. These outcomes could be valuable for scientists in the developing fields of tissue repair and test wound healing.

## Ethical approval

Van Yuzuncu Yil University, Local Ethical Committee of Animal Researches.

Reference number: 2016/03.

Date: 24.03.2016.

## Sources of funding

There is no funding sources.

## Author contribution

Prof. Dr. Nazmi ATASOY: Study design, Data analysis.

Barham Jalal Abdullah: Writing first draft of manuscript.

Abdullah Khalid Omer: Data collection, Critical review and approval of manuscript.

## Conflicts of interest

No conflicts of interest.

## Research registration number

None.

## Guarantor

Prof. Dr. Nazmi ATASOY.

## Research registration unique identifying number (UIN)

Reference number: 2016/03.

Date: 24.03.2016.
