# Supplementary material for: Evaluate the effects of platelet rich plasma (PRP) and zinc oxide ointment on skin wound healing
Source: Ann Med Surg (Lond). 2018 Dec 4;37:30–7. doi: 10.1016/j.amsu.2018.11.009 (PMC6297907; doi:10.1016/j.amsu.2018.11.009)
Supplement: Supplementary data [file mmc1.doc]

**The ARRIVE Guidelines**

**Animal Research: Reporting *In Vivo* Experiments**

|  | **Item** | **Recommendation** |
| --- | --- | --- |
| **TITLE** | **1** | **Evaluate the effects of platelet rich plasma (PRP) and zinc oxide ointment on skin wound healing** |
| **ABSTRACT** | **2** | **In this study efficiency of PRP and zinc oxide on full thickness wounds created on rabbits was researched. This study conducted on 24 New Zealand rabbits divided 3 groups. 1,5 x 1,5cm (2,5cm^2^) full thickness skin wound was created under the general anesthesia. 1ml PRP (5.503106/ mm^3^) was applied to the one of the wounds subcutaneously. To the other wound 1mm^3^ zinc oxide ointment was applied once a day during the study. Wound contraction rates were measured, biopsy materials were collected and evaluated macroscopically and histopathologically postoperatively 3^th^, 7^th^ and 15^th^ days. As a result it is determined that PRP and zinc oxide accelerated wound contraction rates of own groups at rate of p=0.0001. And contraction rates between the groups were determined as 3^th^ day p=0.007, 7^th^ day p=0.0002 and 15^th^ day p=0.002.** |
| **INTRODUCTION** |  |  |
| - **Background** | **3** | 1. **Skin wound healing.** 2. **Wounds are characterized as skin deformities created by electrical, thermal, chemical and mechanical damages that outcome in an opening or damaging the integrity of the skin, or by the occurrence of a fundamental therapeutic or physical issue, or may likewise be characterized as the disturbance of anatomical and physiological integrity of living tissue.** 3. **New Zealand rabbits were selected for this work, skin in human and also in selected experimental species were healed in the same manner and same anatomical and physiological functions.** |
| - **Objectives** |  |  |
| **METHODS** |  |  |
| - **Ethical statement** | **5** | **All experimental protocols were approved by the Van Yuzuncu Yil University, Local Ethical Committee of Animal Researches, Reference number: 2016/03.** |
| - **Study design** | **6** | 1. **Twenty-four New Zealand-white rabbits, were used.** 2. **A 1.5 x 1.5 cm full-thickness skin wound was created under aseptic conditions to reduce or inhibit any infections.** 3. **All animals was divided into two groups based on treatment of wounds, each group containing eight animals.** |
| - **Experimental procedures** | **7** | 1. **Twenty-four New Zealand-white rabbits, weighting about 1.00-1.5 kg with averagely 8 weeks’ old were randomly divided into two groups based on treatment each group containing eight animals.** 2. **All rabbits were anesthetized via intramuscular injection xylazine (3 mg/kg body weight) and ketamine (30 mg/kg body weight) combination. Prior to the experiment, the animals were accustomed to environment about one week and all animals were fasted for 12 hours before the operation.** 3. **Treatment of wounds by PRP and zinc oxide were performed, immediately following the incision wound creation, PRP was used on one wound just once along the wounds time, and zinc oxide was applied to another wounds till the end of selected days in this study based on group treatments.** |
| - **Experimental animals** | **8** | 1. **Twenty-four New Zealand-white rabbits, weighting about 1.00-1.5 kg with averagely 8 weeks’ old were randomly divided into two groups based on treatment each group containing eight animals.** |
| - **Housing and husbandry** | **9** | 1. **All animals were treated in Veterinary Hospital till the end of the work.** 2. **Each animal has a specific cage used for management of rabbits under standard conditions.** |
| - **Sample size** | **10** | 1. **This study conducted on 24 New Zealand rabbits divided 3 groups, each group contains eight animals.** 2. **From each animal, wound contraction rates were measured, biopsy materials were collected and evaluated macroscopically and histopathologically postoperatively 3^th^, 7^th^ and 15^th^ days.** |
| - **Allocating animals to experimental groups** | **11** | **All rabbits were anesthetized via intramuscular injection xylazine (3 mg/kg body weight) and ketamine (30 mg/kg body weight) combination. Prior to the experiment, the animals were accustomed to environment about one week and all animals were fasted for 12 hours before the operation. A 1.5 x 1.5 cm full-thickness skin wound was created under aseptic conditions on waist hip of rabbits. Wound areas were calculated as 2.5 cm^2^ by measuring the edges of the wound at the first day** |
| - **Experimental outcomes** | **12** | **No infection was observed in all animals during the experimental period of this study, and all animals were healthy.** |
| - **Statistical methods** | **13** | **Statistical analyses were performed on graphed prism 6.01 software. ANOVA test was applied to analysis the significant differences in both PRP and zinc oxide groups. Duncan (Kruskal-Wallis) test was done to compare between groups. Differences were considered significant when the P values was =0.0001.**  **Size of wound after healing by both PRP and zinc oxide treatment, during 3^rd^ days (P=0.007), 7^th^ days (P=0.0002) and 15^th^ days (P=0.002).** |
| **RESULTS** |  |  |
| - **Baseline data** | **14** | **Our results indicated a high significant differences (P=0.0001) between PRP and zinc oxide treatments with high significant decreases in size of wound healing. There is a significant difference between PRP treatment and zinc oxide treatment p value=0.007 in third day, also there is significant difference between PRP treatment and zinc oxide treatment p value=0.0002 in seventh days and there is significant difference between PRP treatment and zinc oxide treatment p value= 0.002 in fifteenth days.** |
| - **Numbers analyzed** | **15** | **All groups were analyzed.** |
| - **Outcomes and estimation** | **16** | **This study suggests that topical treatment with autologous PRP can be used as clinical therapy and can enhance tissue healing and enhanced angiogenesis compared to zinc oxide treatments. It can be concluded that PRP results with both macroscopical and microscopical data analysis discovered that wound healing time of PRP therapy group was shorter than that of zinc oxide therapy, and complete re-epithelization was done in both PRP and zinc oxide groups. These outcomes could be valuable for scientists in the developing fields of tissue repair and test wound healing.** |
| - **Adverse events** |  | **There are no adverse events.** |
| **DISCUSSION** |  |  |
| - **Interpretation/scientific implications** | **17** | **Clearly defined in the Section, Discussion.** |
| - **Generalisability/translation** | **18** | **We can use both PRP and zinc oxide to human skin wound healing.** |
| - **Funding** | **19** | **There are no funding sources.** |
